# Photobiomodulation as Nonsurgical Treatment for Crop Burn in *Eupsittula nana* Case Report

**DOI:** 10.1155/crve/1230477

**Published:** 2025-09-19

**Authors:** Diana Cristel Collado Becerril, Luis Eliezer Cruz Bacab, Guadalupe Arjona Jimenez, Eliut Santamaria Mayo

**Affiliations:** Division of Agricultural Sciences, Juarez Autonomous University of Tabasco, Villahermosa, Tabasco, Mexico

**Keywords:** nonsurgical treatment, nursing, PBMT, psittacine

## Abstract

Feeding and nutrition management in young birds is essential for their optimal development. Crop burn and fistula are some of the most common injuries in artificially fed young birds, resulting from the improper use of overheated liquid diets or porridge. A 2-month-old *Eupsittula nana* bird, wild-caught, was presented for consultation with the onset of food leakage through the crop area, which had been present for 1 day. A general physical examination revealed normal physiological parameters, with an alert and responsive attitude, a preserved appetite, and normal-sized bowel movements. A burn fistula was identified in the crop region, with a diameter of approximately 4 mm. Veterinary staff cleaned the fistula with antiseptics prior to photobiomodulation therapy (PBMT) using the PHOVIA system every 48 h on five occasions. The patient received oral meloxicam (1 mg/kg) for 7 days. As a result of the therapy, observed food leakage through the fistula stopped after the third session (sixth day). By the fifth session, the fistula was closed entirely. Without anesthetic management, the use of PBMT represented an efficient tool for the management of crop burns in juvenile birds. Nevertheless, further studies are necessary to validate PBMT effectiveness in crop burning treatment.

## 1. Introduction

Nutritional and feeding management in psittacine birds taken from the wild are highly relevant aspects for their survival in captivity. Crop burn is one of the most common pathologies in these birds [1]. Crop burn occurs when the liquid preparations used for nutritional management are overheated and administered to a bird. This causes a fistula to form, creating a passage from the inside of the crop to the external skin. Caregivers often mistake this lesion for regurgitated residue. The material that escapes through the fistula typically forms a scab, and removing it reveals the underlying defect in the tissue [2, 3]. The method of choice for correcting burn fistulas is reconstructive surgery. However, this procedure carries a high mortality rate, often due to the young age at which the condition presents [2]. In recent years, photobiomodulation therapy (PBMT) has been proposed as a new technique for wound management in companion animals, primarily dogs, cats, and horses [4, 5]. There are no previous reports on the use of PBMT for this condition in nonconventional companion animals such as psittacines.

## 2. Case Presentation

**Figure 1:**
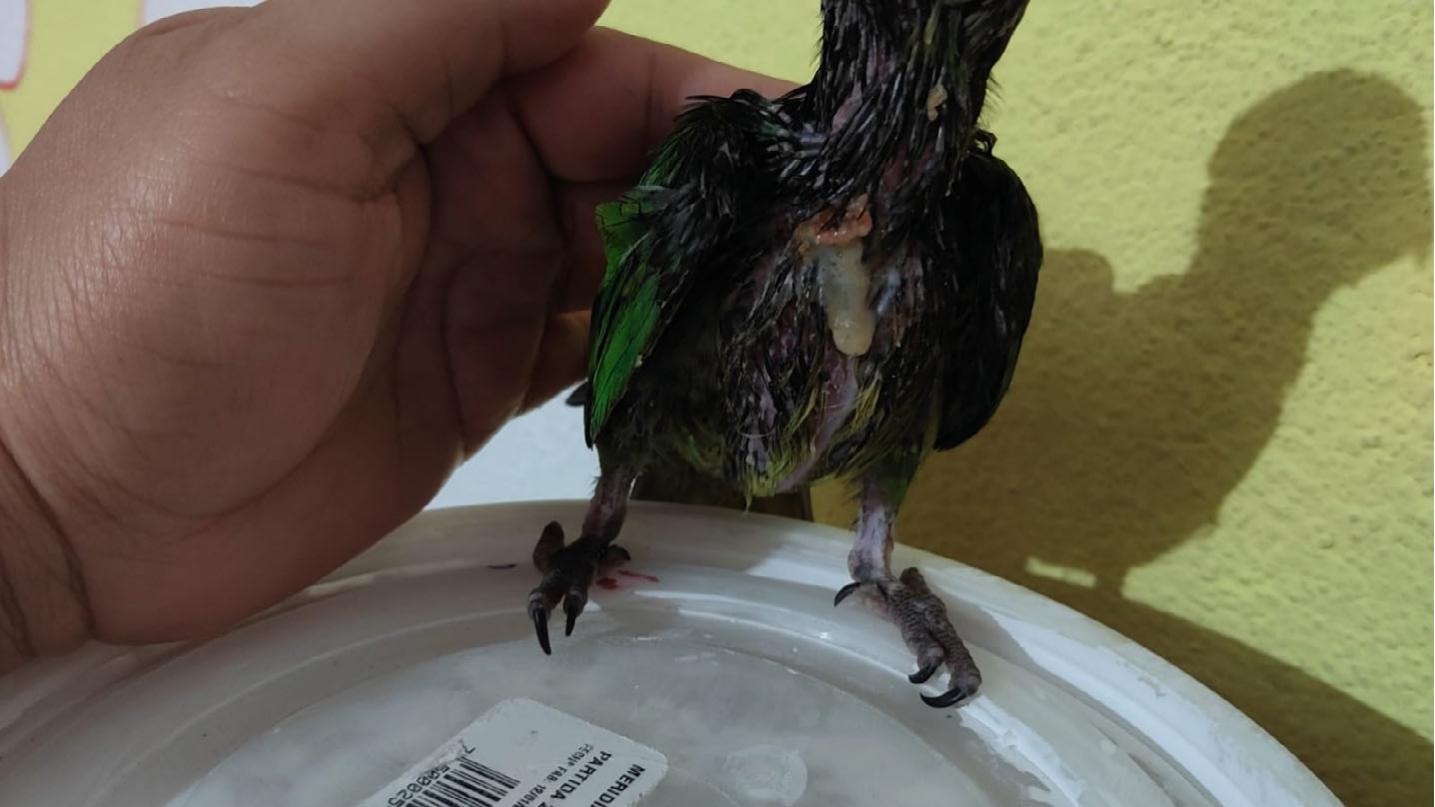
Day after crop burn.

**Figure 2:**
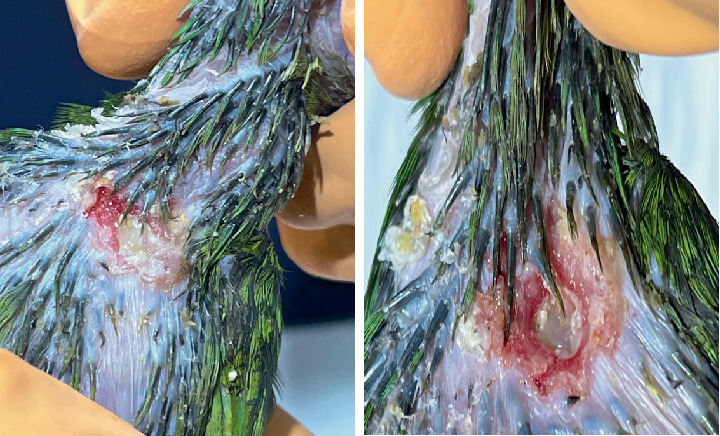
Crop burn fistula.

A 60-day-old *Eupsittula nana* psittacine was presented for consultation after being removed from a home. A “scab” had been observed on the crop area after the bird was fed warm porridge (Figures 1 and 2). The patient weighed 78 g, had a normal gait, regular bowel movements, and maintained an appetite despite the lesion. The patient is kept loose in the home by its owners and lives with two other psittacines of different species.

### 2.1. Fistula Cleaning

Prior to PBMT, veterinary staff cleaned the fistula area with Veteribac antiseptic, followed by Prontosan using sterile gauze to remove the crust of food adhering to the outer skin. The crust was then removed from the fistula area, which was subsequently dried with a cotton swab. The fistula measured approximately 4 mm, which was the same size as the head of the cotton swab. Meloxicam was administered intramuscularly at 1 mg/kg of body weight into the breast muscle. For home treatment, oral meloxicam at 1 mg/kg was recommended for 7 days.

### 2.2. PBMT

**Figure 3:**
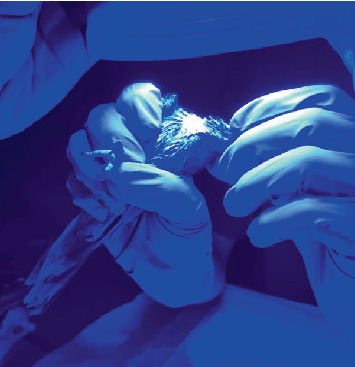
PBMT-PHOVIA system.

The therapy consisted of five sessions. Each session was divided into two applications administered every 48 h. The sessions involved the initial application of a thin layer of the chromophore-based gel, which was composed of the following ingredients: water, glycerin, propylene glycol, and urea peroxide, followed by the use of the PHOVIA lamp. The PHOVIA lamp emits noncoherent blue light at a single-peak wavelength, with a peak emission between 440 and 460 nm. Its power density ranges from 55 to 129 mW/cm^2^ at 5 cm on a 50 cm^2^ illuminated round surface. The lamp is equipped with a 2-min timer. The distance between the lamp and the fistula was approximately 5 cm. Cleaning of the chromophore-based gel was performed using a sterile gauze after each application (Figure 3). The second application was performed in the same manner, with a 2-min interval between the two applications of each session.

**Figure 4:**
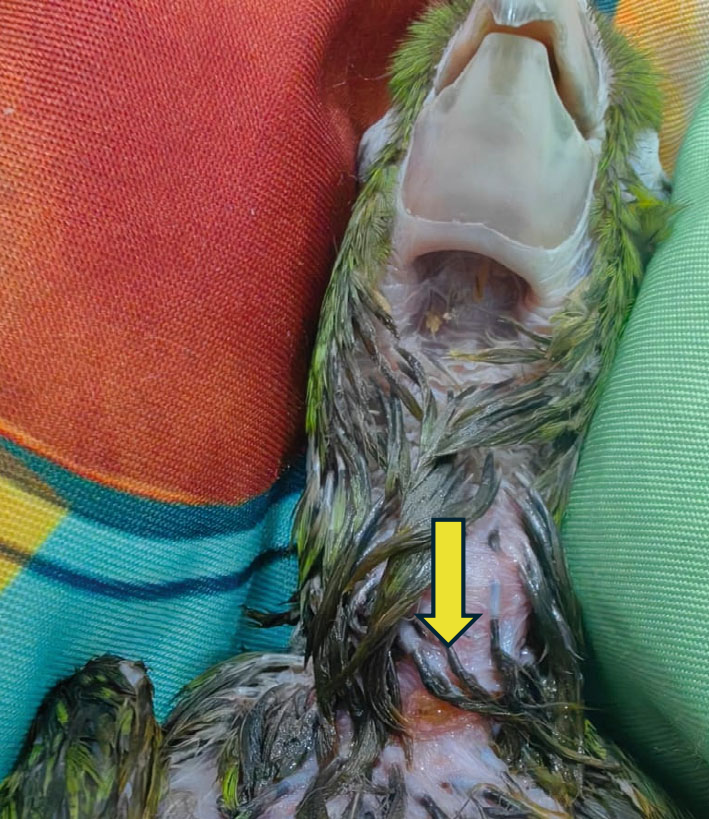
Result after the fifth PBMT session.

**Figure 5:**
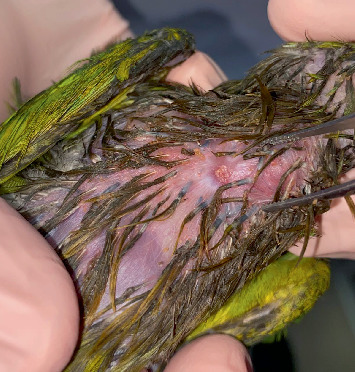
Final result after PBMT.

During the treatment period, the patient was managed at home with feeding recommendations. The feeding was divided into several meals during the day to avoid leakage until the fistula healed. At home, the patient kept loose by the caregivers (Figures 4 and 5).

### 2.3. Ethical Aspects

In Mexico, the species *Eupsittula nana* is included in the list of protected species under the official Mexican standard NOM-059-SEMARNAT-2010. This standard establishes risk categories for native species and specifies the criteria for their inclusion in, or exclusion from, protected lists. Therefore, keeping these birds as pets is not legally permitted. However, birds presented for medical care can be treated by veterinarians without restrictions. Additionally, the Ministry of Agriculture and Rural Development (SADER by its acronym in Spanish) issues health regulations for the prevention and control of diseases, particularly in birds, including Psittacidae.

Regarding the patient's caregivers, we requested their authorization to use the information generated from their bird's clinical case and treatment. The caregivers expressly authorized the use of the information, without including any data protected by Mexico's Federal Law on the Protection of Personal Data held by private parties.

## 3. Discussion

The crop is a significant dilation of the esophagus that serves as a temporary food storage site. It is located just before the esophagus enters the thoracic cavity. Problems with the crop most frequently occur in neonatal and young birds [1]. Crop burns are observed in juvenile birds, usually psittacines, following gavage feeding of improperly warmed formula that causes thermal injury to the crop mucosa. Overheated or poorly stirred formula can result in hot areas within the mixture, leading to thermal burns and subsequent mucosal and skin necrosis. A scab forms over the affected area and eventually lifts away. Other possible causes of crop burn include injuries from animal bites, chronic irritations from chemical substances, or foreign bodies such as sharp iron objects. Although crop burn is typically described in juvenile birds, it can also occur in adult birds. In both juvenile and adult birds, crop burns can result in significant loss of crop tissue, which compromises adequate feeding and nutrition [2, 3, 6–8]. Damaged skin and crop tissues must be debrided and sutured as separate layers. Closing the wound requires dermoplastic techniques, although healing by secondary intention is also an option [2, 6, 9]. According to Jenkins [2], Kumar et al. [10], and Miles [11], avian surgeons must have a thorough understanding of the anatomical and physiological characteristics of birds. To perform successful surgical procedures in avian patients, a complete clinical examination and stabilization should be carried out. All surgical materials and instruments must be readily available to ensure the surgeon's work is efficient. It is also a priority to consider equipment that will maintain the patient's stability during surgery. Magnification tools, proper lighting, and appropriately sized suture materials can all promote surgical success.

According to Seamon et al. [12], the mortality rate associated with inhalational anesthesia is up to 7.7% in avian patients, which is higher than in feline, canine, and human patients. Despite the use of standardized anesthetic protocols and appropriate equipment, the possibility of death still exists for the avian patient.

On the other hand, Ashton et al. [13] point out that age, sex, and weight have no significant impact on the odds of death during or within 48 h of general anesthesia cessation. According to Barron [14], wounds are a common and debilitating reason for care in wild birds. When wounds do not heal promptly, there is an increase in discomfort and disease that interferes with health and quality of life. This can lead to the development of new problems and may affect the outcome of the case. For wild birds that must have appropriate function for their wild lifestyle after recovery, permanent disability or delayed healing may become an indication for euthanasia.

Alternative techniques for crop burning treatment have been explored, such as removing debris from the wound before using topical medications. The use of topical antibiotics is indicated when treating infected wounds. Ointments may initially help to keep tissues moist, but they can also delay wound healing by impeding the migration of fibroblasts into the area. Knapp-Hoch and de Matos [15] reported that another technique is vacuum-assisted closure (VAC), which may reduce healing time, but there are scarce publications on its adaptation for birds. External coaptation and physical therapy, including the use of bandages, can serve as temporary management in emergent situations. Nevertheless, it is important to mention that anesthesia or sedation may be necessary for adequate wound care using these alternative techniques, which is not required with PBMT [14].

Therefore, the use of PBMT or light therapy represents an effective alternative for the management of crop burn, without the need for anesthesia in the avian patient. Light therapy dates to ancient civilizations [4, 16]. The North American Association for Light Therapy and the World Association for Laser Therapy agreed by consensus in 2014 to promote the term PBMT. They defined it as a form of light treatment that uses nonionizing light sources, including lasers, light-emitting diodes (LEDs), and broadband light, in the visible and infrared spectrum. This nonthermal process involves endogenous chromophores that elicit photophysical (i.e., linear and nonlinear) and photochemical events at various biological scales. However, several other uses for PBMT are being actively investigated for specific anatomical pathologies (e.g., wounds on limbs vs. mucosa) or disease-specific pathologies (e.g., venous vs. diabetic wounds).

Regarding companion animals such as rodents, rabbits, canines, and equines, Anders et al. [17], Arany [18], Hochman [19], and Lopez and Brundage [5] report that PBMT results in beneficial therapeutic outcomes. These include pain or inflammation alleviation, immunomodulation, and promotion of wound healing and tissue regeneration. For these reasons, Pryor and Millis [20] reported that PBMT is a popular treatment alternative in both general and specialty veterinary hospitals in North America. Nevertheless, its use in avian patients is still scarce.

## 4. Conclusion

In the present case, the patient showed significant improvement after the third session of PBMT. By the fifth session, the fistula closed completely. The use of PBMT represents an efficient tool for the management of crop burns in juvenile birds without anesthetic management. Nevertheless, further studies are necessary to validate PBMT's effectiveness in crop burning treatment.

## Data Availability

The data that support the findings of this study are available from the corresponding author upon reasonable request.
